# Assessing the Effects of Redox Modifier MnTnBuOE-2-PyP 5+ on Cognition and Hippocampal Physiology Following Doxorubicin, Cyclophosphamide, and Paclitaxel Treatment

**DOI:** 10.3390/ijms21051867

**Published:** 2020-03-09

**Authors:** Taylor McElroy, Taurean Brown, Fred Kiffer, Jing Wang, Stephanie D. Byrum, Rebecca E. Oberley-Deegan, Antiño R. Allen

**Affiliations:** 1 Division of Radiation Health, University of Arkansas for Medical Sciences, Little Rock, AR 72205, USA; TMMCELROY@uams.edu (T.M.); TBrown8@uams.edu (T.B.); KIFFERF@EMAIL.CHOP.EDU (F.K.); JWang2@uams.edu (J.W.); 2 Department of Pharmaceutical Sciences, University of Arkansas for Medical Sciences, Little Rock, AR 72205, USA; 3 Neurobiology & Developmental Sciences, University of Arkansas for Medical Sciences, Little Rock, AR 72205, USA; 4 Department of Biochemistry and Molecular Biology, University of Arkansas for Medical Sciences, Little Rock, AR 72205, USA; SBYRUM@uams.edu; 5 Arkansas Children’s Research Institute, Little Rock, AR 72202, USA; 6 Department of Biochemistry and Molecular Biology, University of Nebraska Medical Center, Omaha, NE 68198, USA; becky.deegan@unmc.edu

**Keywords:** doxorubicin, cyclophosphamide, paclitaxel, MnTnBuOE-2-PyP, hippocampus, cognition

## Abstract

Background: Chemotherapy treatment for breast cancer can induce cognitive impairments often involving oxidative stress. The brain, as a whole, is susceptible to oxidative stress due to its high-energy requirements, limited anaerobic respiration capacities, and limited antioxidant defenses. The goal of the current study was to determine if the manganese porphyrin superoxide dismutase mimetic MnTnBuOE-2-PyP (MnBuOE) could ameliorate the effects of doxorubicin, cyclophosphamide, and paclitaxel (AC-T) on mature dendrite morphology and cognitive function. Methods: Four-month-old female C57BL/6 mice received intraperitoneal injections of chemotherapy followed by subcutaneous injections of MnBuOE. Four weeks following chemotherapy treatment, mice were tested for hippocampus-dependent cognitive performance in the Morris water maze. After testing, brains were collected for Golgi staining and molecular analyses. Results: MnBuOE treatment preserved spatial memory during the Morris water-maze. MnBuOE/AC-T showed spatial memory retention during all probe trials. AC-T treatment significantly impaired spatial memory retention in the first and third probe trial (no platform). AC-T treatment decreased dendritic length in the Cornu Ammonis 1 (CA1) and dentate gyrus (DG) areas of the hippocampus while AC-T/MnBuOE maintained dendritic length. Comparative proteomic analysis revealed affected protein networks associated with cell morphology and behavior functions in both the AC-T and AC-T/MnBuOE treatment groups.

## 1. Introduction

During the last 20 years, breast cancer (BC) patients have self-reported cognitive impairments after chemotherapy bringing awareness to both the clinical and scientific communities [1,2]. Chemotherapy-induced cognitive impairment (CICI) or more commonly known as “chemobrain” is characterized by deficits in memory, learning, attention, concentration, visuospatial skills, and executive functioning [3]. Prevalence is hard to quantify, but an estimated range of 14–85% of cancer patients are thought to be affected during and after chemotherapy treatment [4]. This research will focus on one common breast cancer chemotherapy regimen: doxorubicin, cyclophosphamide, and paclitaxel (AC-T). In the United States, breast cancer is the most prevalent cancer amongst women, with an estimated 5 million cases by 2030 [5,6]. As the number of cases increases, the survivorship also increases due to an aging population, and advancements in early detection, and treatment. As BC patients live longer post-chemotherapy, the long-term side effects of the treatment emerge: chemobrain can persist 10–20 years after treatment.

Adjuvant chemotherapy is used to treat both early and late-stage breast cancer [7]. For breast cancer patients without pre-existing heart disease, standard treatment evolved to include polychemotherapy using an anthracycline-containing regimen. Four cycles of doxorubicin and cyclophosphamide (AC) followed by four cycles of paclitaxel (T) became standard treatment (AC-T) in the 1990s due to its modest toxicity profile and improvement in disease-free survival [8,9]. Chemotherapy either directly or indirectly causes oxidative stress, which can lead to failure of normal cell function. Oxidative stress, wherein the generation of reactive species exceeds a cell’s adaptive and repair capacities, is considered one of the potential mechanisms behind chemobrain [10]. Doxorubicin cannot cross the blood-brain barrier (BBB) or the microvasculature of the central nervous system (CNS) that regulates movement of molecules, ions, and cells between the blood and CNS [11]. Despite this, doxorubicin indirectly causes oxidative stress in the brain via production of the cytokine tumor necrosis factor alpha (TNF-⍺) which can permeate the BBB. Doxorubicin has been shown in rodent models to decrease levels of glutathione, decrease glutathione S-transferase activity, and increase nitric oxide synthase (NOS) activity in the brain, all of which could reflect mechanisms of oxidative stress [12,13]. Cyclophosphamide has also been shown to increase oxidative stress in the rodent central nervous system (CNS) by increased malondialdehyde levels [14], inhibition of brain catalase, and decreased glutathione levels [14,15,16]. 

Over the last 30 years, small molecule mimics of the superoxide dismutase (SOD) enzyme have been developed and employed to decrease normal tissue injury by acting as a redox modulator [17]. MnTnBuOE-2-PyP 5+ (MnBuOE) is a third generation, cationic, lipophilic, manganese porphyrin (MnP) molecule. As a porphyrin-based mimetic of the human mitochondrial manganese SOD, it is a potent mimetic [18,19]. It is able to cross the BBB [18] and protect the brain against oxidative damage. MnBuOE has a higher therapeutic potential than earlier generations of MnPs. Besides superoxide, MnPs can react with other reactive oxygen, nitrogen, and sulfur species [20]. Depending on the environment, MnPs can act in either an antioxidant or pro-oxidant capacity. MnBuOE has been shown to sensitize tumor cells to chemotherapy and radiotherapy while protecting normal tissue by modulating tissue redox [17,21].

Data obtained from our laboratory and others have demonstrated that chemotherapy adversely affects adult murine learning and memory in addition to altering hippocampal morphology. Specifically, Thiotepa administration in adult male mice caused deficits in short-term memory and in spatial memory retention [22]. 5-FU administrations compromised the structure of neurons in the hippocampus as seen by decreased dendritic length, complexity, and spine density [23]. Seigers et al. showed that administration of cyclophosphamide or doxorubicin in adult male mice resulted in deficits in novel location recognition, a hippocampal dependent memory task. The hippocampus is crucial for the consolidation of short-term to long-term memories, declarative memories, and spatial navigation [24,25]. Although investigating the effects of individual chemotherapeutic agents offers insights into mechanism behind chemobrain, cancer treatments are commonly administered in combination including doxorubicin, cyclophosphamide, and paclitaxel (AC-T). Knowing that the individual chemotherapies in this regimen cause oxidative stress in the CNS, it is the goal of this study to see if MnBuOE, used as a redox modifier, can ameliorate the effects of AC-T chemotherapy on cognition and hippocampal morphology.

## 2. Results

### 2.1. Morris Water Maze (MWM)

#### 2.1.1. Distance Moved

**Figure 1:**
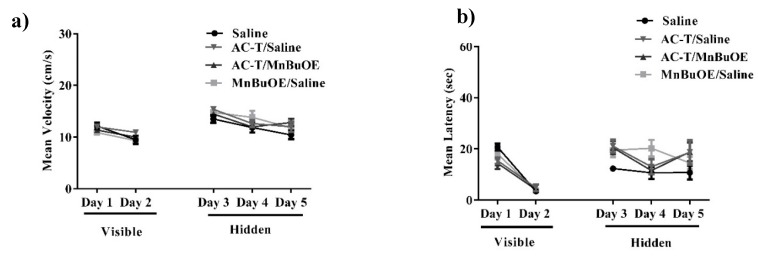
Velocity and latency to the target platform during visible and hidden training sessions. (**a**,**b**) There were no significant differences in latency or distance moved between the treatments throughout testing. Mean ± SEM (*n* = 8).

Hippocampal-dependent spatial learning and memory were assessed with the 5-day MWM. Improvement in spatial learning and memory is represented by a decrease in path latency (i.e., time) to reach the platform. A two-way repeated measures ANOVA of velocity revealed significant differences in treatment-by-day interactions (F _(4,112)_ = 27.21; *p* < 0.0001; Figure 1a), but Holm’s correction revealed that there were no significant differences between treatments on either training day. An ordinary two-way ANOVA of latency revealed no significant differences in treatment effect (F _(3,140)_ = 2.24; *p* = 0.09; Figure 1b). There were significant differences in treatment-by-day interactions (F _(4,140)_ = 23.15; *p* < 0.0001; Figure 1b) meaning mouse performance of the test improved as testing progressed which suggests that the AC-T does not affect the ability of mice to learn during the MWM task.

#### 2.1.2. Probe Trials

**Figure 2:**
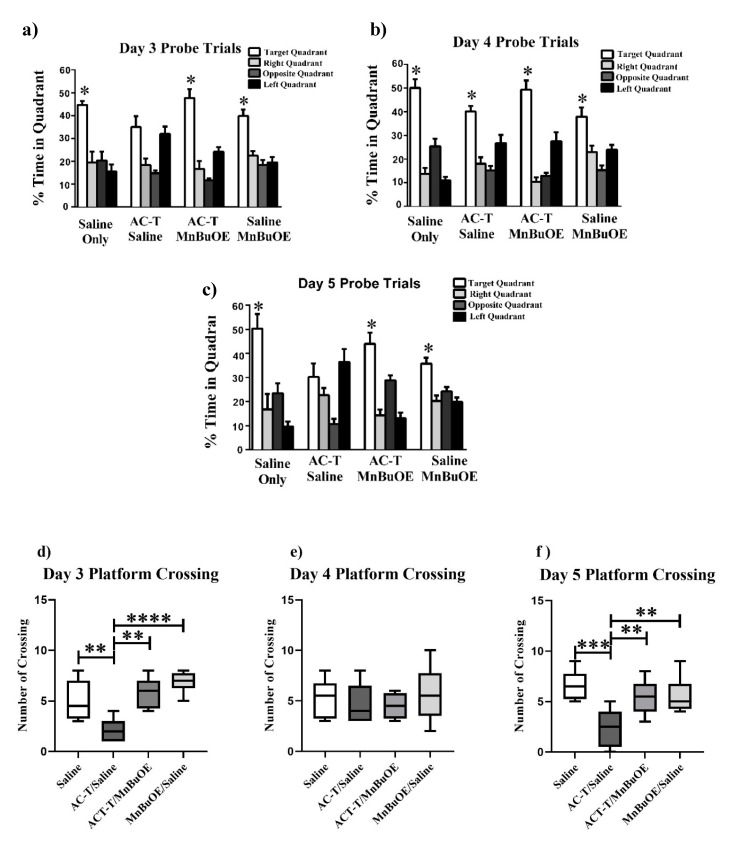
Spatial memory retention during probe trials on days 3–5 of Morris water maze testing. (**a**) The saline, MnBuOE, and AC-T/MnBuOE groups spent significantly more time in the target quadrant than other quadrants. (**b**) All treatment groups spent significantly more time in the target quadrant than other quadrants. (**c**) The saline, MnBuOE, and AC-T/MnBuOE groups spent significantly more time in the target quadrant than other quadrants. (**d**–**f**) After removal of the platform on day 3–5, the number of platform crossings. Each bar represents the mean of 8 mice; error bars are the SEM. *p < 0.05, **p < 0.01, ***p < 0.001, ****p < 0.0001.

To assess spatial memory retention, probe trials were done where the platform was removed after the hidden platform training on days 3–5. Normal, non-impaired animals demonstrate spatial memory retention by spending more time in the target quadrant as compared to the other quadrants. During the first probe trial on day 3, significant differences were revealed for the saline treated group (F _(3,28)_ = 14.34, *p* < 0.0001; Figure 2a), the AC-T/MnBuOE treated group (F _(3,28)_ = 31.09, *p* < 0.0001; Figure 2a), and the saline/MnBuOE group (F _(3,28)_ = 18.10, *p* < 0.0001; Figure 2a). The AC-T treated mice were able to discriminate between the target, right, and opposite quadrants on day 3 (F _(3,28)_ = 9.35, *p* < 0.001; Figure 2a). However, Holm’s correction revealed there was no significant difference between the target and left quadrant (*p* = 0.51, Figure 2a). During the second probe trial on day 4, all treatment groups showed a significant difference in preference for the target quadrant: saline (F _(3,28)_ = 39.80, *p* < 0.0001; Figure 2b), AC-T (F _(3,28)_ = 17.96, *p* < 0.0001; Figure 2b), AC-T/MnBuOE (F _(3,28)_ = 37.84, *p*< 0.0001; Figure 2b), and saline/MnBuOE (F _(3,28)_ = 11.89, *p* < 0.0001; Figure 2b). Similar to the first probe trial results, the results for the third probe trial on day 5 revealed significant differences for the saline treated group (F _(3,28)_ = 12.77, *p* < 0.0001; Figure 2c), the AC-T/MnBuOE-treated group (F _(3,28)_ = 22.16, *p* < 0.0001; Figure 2c), and the saline/MnBuOE-group (F _(3,28)_ = 12.55, *p* < 0.0001; Figure 2c). The AC-T treated mice were able to discriminate between the target, right, and opposite quadrants on day 5 (F _(3,28)_ = 6.67, *p* < 0.01; Figure 2c). However, Holm’s correction revealed there was no significant difference between the target and left quadrant (*p* = 0.40, Figure 2c) demonstrating a memory impairment. We have calculated platform crossing in the first probe trial of the water maze test. We compared all treated mice and found there was a significant difference day 3 and 5 between the treatment groups (Figure 2d, f). Following a second day of hidden platform training (Probe 2), all groups showed memory retention and spent more time searching in the target quadrant than in any other quadrant (Figure 2e).

### 2.2. Dendritic Morphology

#### 2.2.1. Dentate Gyrus (DG)

**Figure 3:**
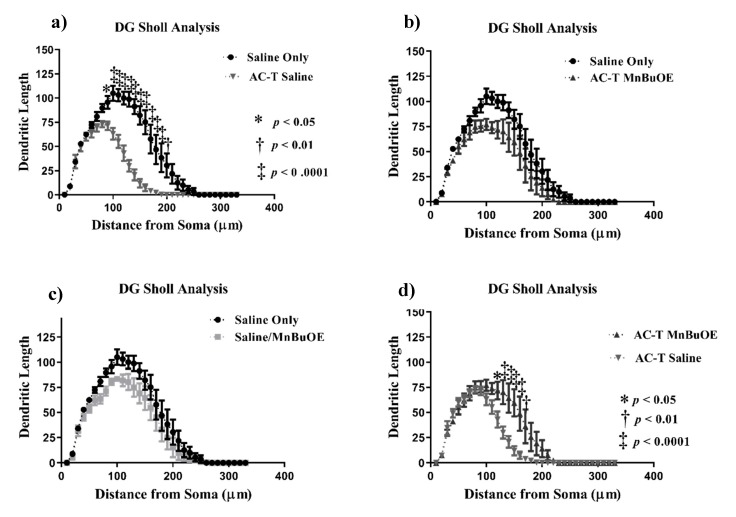
Sholl analysis throughout the DG region of the hippocampus. (**a**) AC-T treatment significantly decreased dendritic length as compared to saline treatment. (**b**) AC-T/MnBuOE treatment did not affect dendritic length. (**c**) MnBuOE treatment did not affect dendritic length. (**d**) AC-T chemotherapy significantly decreased dendritic length as compared to AC-T/MnBuOE treatment. Mean ± SEM (*n* = 8); * *p* < 0.05; ** *p* < 0.01; ‡ *p* < 0.0001.

In order to investigate the effects of the AC-T regimen on dendritic length, Sholl analysis was performed looking at dendritic length as a function of increasing 10 µm intervals from the soma. In the DG, there was a significant interaction between treatment and dendritic length (F = 8.514, *p* = 0.0013; Figure 3), dendritic complexity (F = 3.57, *p* < 0.05; Figure 6a), nodes (F = 6.34, *p* < 0.01), end points (F = 6.75, *p* < 0.01), and surface area (F = 9.691, *p* < 0.001) that was significant after Holm–Sidak post-hoc analysis. The two-way repeated measures ANOVA also detected a main effect of treatment (F _(1,4)_ = 28.98, *p* = 0.0058; Figure 3) and length (F _(32,128)_ = 83.01, *p* < 0.0001; Figure 3a). Post-hoc analysis revealed that AC-T-treated mice had decreased dendritic length at 100–180 µm (Sidak, *p* < 0.0001), 190 µm (*p* = 0.0001), and 200 µm (*p* = 0.0057).

After Holm–Sidak multiple comparisons, MnBuOE treatment did not show a significant interaction between treatment and dendritic length, dendritic complexity, nodes, end points, or surface area. There was a main effect of treatment (F _(1,4)_ = 13.2 *p* = 0.0221; Figure 3) and distance (F _(32,128)_ = 57.33, *p* < 0.0001; Figure 3c). Post-hoc analysis revealed significant changes in dendritic length at 130 µm (Sidak, *p* = 0.0308). AC-T/MnBuOE treatment as compared with saline alone did show a significant interaction between treatment and dendritic length (F =8.514, *p* = 0.0013; Figure 3), end points (F = 6.75, *p* < 0.01), and surface area (F = 9.69, *p* < 0.001) that remained significant after Holm-Sidak analysis. There was a main effect of Sholl length (F _(1.55,6.19)_ = 0.0484 *p* = 0.0001: Figure 3b). Post-hoc analysis revealed no significant changes in dendritic length.

When comparing AC-T-treated with ACT/MnBuOE-treated mice, there were no significant interactions between treatment and dendritic length, dendritic complexity, nodes, end points, or surface area after Holm-Sidak analysis (Figure 3). There was a main effect of treatment (F _(1, 4)_ = 16.07, *p* = 0.016; Figure 3) and distance (F _(32,128)_ = 48.02, *p* < 0.0001; Figure 3d). Post-hoc analysis revealed that AC-T/MnBuOE-treated mice had increased dendritic length at 120 µm (Sidak, *p* = 0.0123), 130–160 µm (*p* < 0.0001), and 170 µm (*p* = 0.0027) from the soma. Dendritic spine density was quantified for different types of spines. We found no significant changes in thin spines (F _(3, 36)_ = 1.51, *p* = 0.23) or stubby spines (F _(3, 36)_ = 1.00, *p* = 0.40). MnBuOE treatment significantly increased mushroom spine density (F _(3, 36)_ = 3.22 *p* < 0.05; Figure 7a).

#### 2.2.2. CA1 Pyramidal Neurons

**Figure 4:**
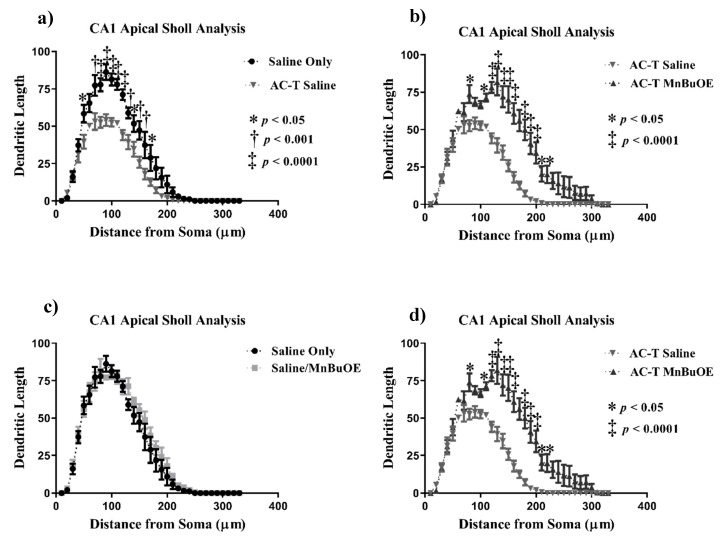
Sholl analysis throughout the apical CA1 region of the hippocampus. (**a**) AC-T treatment significantly decreased dendritic length as compared to saline treatment. (**b**) AC-T/MnBuOE treatment significantly increased dendritic length. (**c**) MnBuOE treatment did not affect dendritic length. (**d**) AC-T chemotherapy significantly decreased dendritic length as compared to AC-T/MnBuOE treatment. Mean ± SEM (*n* = 8); * *p* < 0.05; ** *p* < 0.01; ‡ *p* < 0.0001.

Similar analyses were done with the apical and basal regions of the CA1 region. In the apical CA1 neurons, AC-T treatment resulted in significant interaction between treatment and dendritic length (F = 17.79, *p* < 0.0001; Figure 4), dendritic complexity (F _(3, 16)_ = 8.931, *p* < 0.01; Figure 6b), nodes (F _(3, 16)_ = 13.06, *p* < 0.001), end points (F _(3, 16)_ = 12.28, *p* < 0.001), and surface area (F _(3, 16)_ = 18.62, *p* < 0.0001). After Holm–Sidak multiple comparison analysis, the interactions were still significant. The two-way repeated measures ANOVA also detected a main effect of treatment (F _(1, 4)_ = 66.47, *p* = 0.0012; Figure 4) and distance (F _(32, 128)_ = 108.2, *p* < 0.0001; Figure 4a). Post-hoc analysis revealed that AC-T-treated mice had decreased dendritic length at 50 µm (Sidak, *p* = 0.0119), 70 µm (*p* = 0.0002), 80–120 µm (*p* < 0.0001), 130 µm (*p* = 0.0074), 140 µm (*p* = 0.0334), and 150–160 µm (*p* < 0.01).

After Holm-Sidak multiple comparisons, MnBuOE treatment did not show a significant interaction between treatment and dendritic length, dendritic complexity, nodes, end points, or surface area (Figure 4). There was a main effect of distance (F _(32, 128)_ = 119.3, *p* < 0.0001; Figure 4c). Post-hoc analysis revealed no significant changes in dendritic length. When compared to AC-T alone, MnBuOE showed a significant interaction between treatment and dendritic complexity that was significant after multiple comparisons (*p* < 0.01; Figure 6b). AC-T/MnBuOE treatment did not show a significant interaction between treatment and dendritic length, dendritic complexity, nodes, end points, or surface area after Holm-Sidak multiple comparisons (Figure 4). There were main effects of distance (F _(2.837,11.35)_ = 92.65, *p* < 0.0001; Figure 4c). Post-hoc analysis revealed no significant changes in dendritic length.

Looking at the comparison between the AC-T-treated mice and the AC-T/MnBuOE-treated mice there was a significant interaction after Holm-Sidak post-hoc analysis between treatment and dendritic length, dendritic complexity (*p* < 0.001; Figure 6b), nodes, end points, and surface area. There were main effects of treatment (F _(1, 4)_ = 37.81, *p* =0.0035; Figure 4) and distance (F _(1.646,6.583_ = 89.48, *p* < 0.0001; Figure 4d). Post-hoc analysis revealed that the addition of MnBuOE to the AC-T regimen increased dendritic length at160 µm (Sidak, *p* = 0.0168). For dendritic spine density there were no significant changes in thin spines (F _(3, 16)_ = 1.382, *p* = 0.28) or stubby spines (F _(3, 16)_ = 2.120, *p* = 0.137). MnBuOE treatment alone significantly increased mushroom spine density (F _(3, 16)_ = 8.055, *p* < 0.01; Figure 7b).

**Figure 5:**
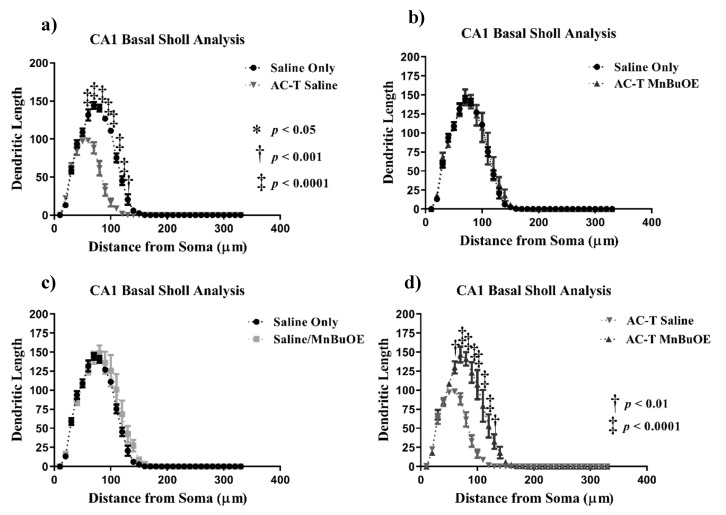
Sholl analysis throughout the basal CA1 region of the hippocampus. (**a**) AC-T treatment significantly decreased dendritic length as compared to saline treatment. (**b**) AC-T/MnBuOE treatment did not affect dendritic length. (**c**) MnBuOE treatment did not affect dendritic length. (**d**) AC-T chemotherapy significantly decreased dendritic length as compared to AC-T/MnBuOE treatment. Mean ± SEM (*n* = 8); * *p* <0.05; ** *p* < 0.01; ‡ *p* < 0.0001.

**Figure 6:**
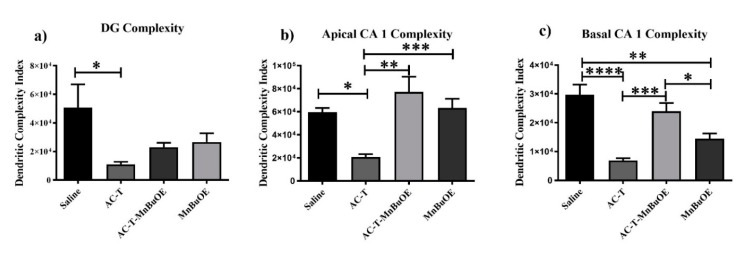
Dendritic complexity throughout the hippocampus. (**a**) AC-T treatment significantly reduced complexity in the dentate gyrus (DG). (**b**,**c**) AC-T treatment significantly reduced dendritic complexity in the apical and basal CA1 region. Mean ± SEM (*n* = 8); * *p* < 0.05, ** *p* < 0.01, *** *p* < 0.001; **** *p* < 0.0001.

In the basal CA1 neurons, AC-T treatment produced a significant interaction between treatment and dendritic length (F = 20.87, *p* < 0.0001; Figure 5), dendritic complexity (F = 16.44, *p* < 0.0001; Figure 6c), nodes (F = 16.25, *p* < 0.0001), end points (F = 15.39, *p* < 0.0001), and surface area (F = 16.97, *p* < 0.0001). After Holm–Sidak multiple comparisons, the interactions were still significant. There were main effects of treatment (F _(1, 4)_ = 114.2, *p* = 0.0004; Figure 5) and distance (F _(2.108,8.433)_ = 313.7, *p* < 0.0001; Figure 5a). Post-hoc analysis revealed AC-T decreased dendritic length at 90 µm (Sidak, *p* = 0.0203), 100 µm (*p* = 0.0055) and 110 µm (*p* = 0.0406). MnBuOE treatment showed a significant interaction between treatment and dendritic complexity (F = 16.44, *p* < 0.0001; Figure 6c). After the Holm–Sidak post-hoc analysis, the differences between length, nodes, end points, and surface area were not significant. There was a main effect of distance (F _(32,128)_ = 171.9, *p* < 0.0001; Figure 5c). Post-hoc analysis revealed MnBuOE significantly increased dendritic length at 110 µm (Sidak, *p* = 0.0293). After Holm-Sidak multiple comparisons, AC-T/MnBuOE treatment did not show a significant interaction between treatment and dendritic length, dendritic complexity, nodes, end points, or surface area. There was a main effect of distance (F _(32, 128)_ = 155.1, *p* < 0.0001; Figure 5b). Post-hoc analysis did not reveal significant changes in Sholl length.

**Figure 7:**
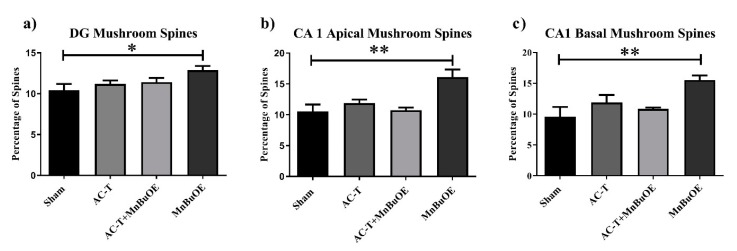
Mushroom spines throughout the hippocampus. (**a**–**c**) MnBuOE treatment significantly increased the number of mushroom spines in the apical and basal regions of the CA1 and in the DG. Mean ± SEM (*n* = 8); * *p* <0.05; ** *p* < 0.01.

Looking at the comparison between the AC-T-treated mice and the AC-T/MnBuOE-treated mice in the basal CA1, there were significant interactions between treatment and dendritic length, dendritic complexity (*p* < 0.001; Figure 6c), nodes, end points, and surface area even after Holm–Sidak post-hoc analysis. There were main effects of distance (F _(32,128)_ = 116.3, *p* < 0.0001; Figure 5d) and treatment (F _(1, 4)_ = 30.30, *p* = 0.0053; Figure 5). After post-hoc analysis, MnBuOE in addition to AC-T significantly increased dendritic length at 60 µm (Sidak, *p* = 0.0028), 70–120 µm (*p* < 0.0001) and at 130 µm (*p* = 0.0022). For dendritic spine density there were no significant changes in thin spines (F _(3, 16)_ = 1.189, *p* = 0.3454) or stubby spines (F _(3, 16)_ = 2.228, *p* = 0.1244). MnBuOE treatment alone significantly increased mushroom spine density (F _(3, 16)_ = 5.79, *p* < 0.01; Figure 7c).

### 2.3. Proteomics

A set of 211 proteins were identified as differentially expressed between saline-treated and chemotherapy (AC-T) treated mice (Table 1 and Table S1). The proteins were annotated with their respective cellular localization and protein type. For the comparison between AC-T treated mice and AC-T/MnBuOE treated mice, a set of 147 proteins was identified as differentially expressed (Table 2 and Table S1).

**Figure 8:**
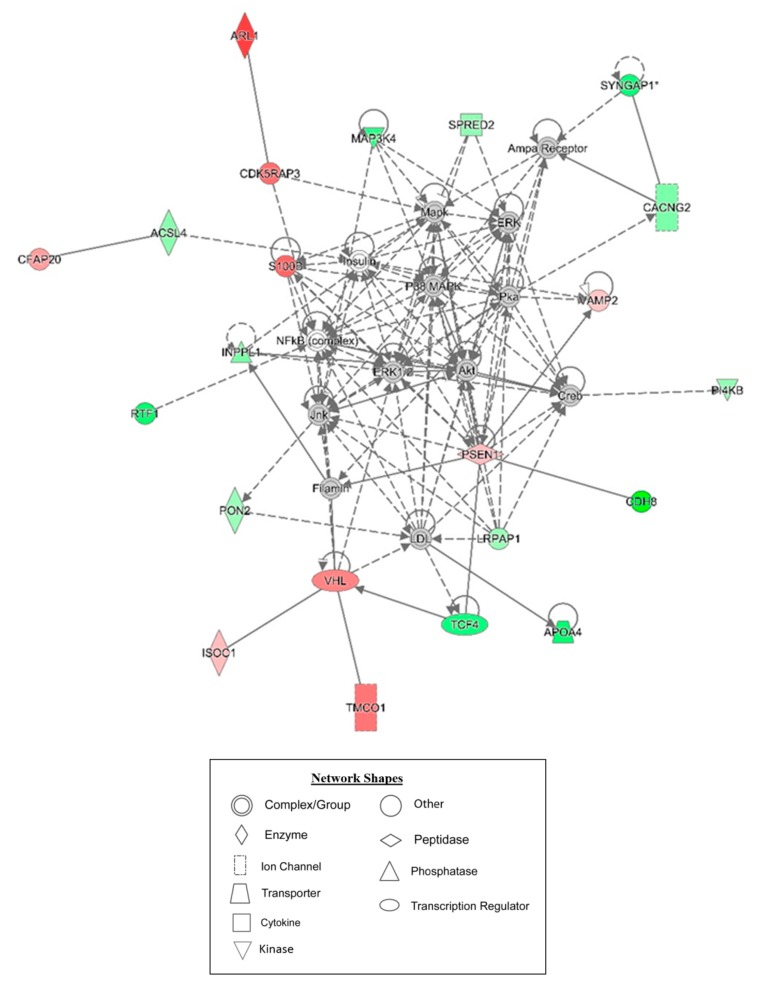
Graphic representation of mouse hippocampus protein network 1, identified by IPA as being affected by AC-T treatment. Functions associated with the network include neurological disease, cell morphology, and cell-to-cell signaling and interaction. The color of the node depicts differential expression. Red represents upregulated proteins. Green represents downregulated proteins. The intensity of the color denotes the degree of regulation where brighter colors are more regulated. Gray node color reflects proteins that were found in the data set but were insignificant expression wise. Uncolored nodes were not identified as differentially expressed in our data, but were incorporated into the computational network based on evidence stored in the Ingenuity Knowledge Base. Known direct and indirect interactions between network proteins, as well as the direction of the interaction, are indicated by arrows or blocked lines. Central to the network are p38 MAPK, Akt, Jnk, and Creb.

**Figure 9:**
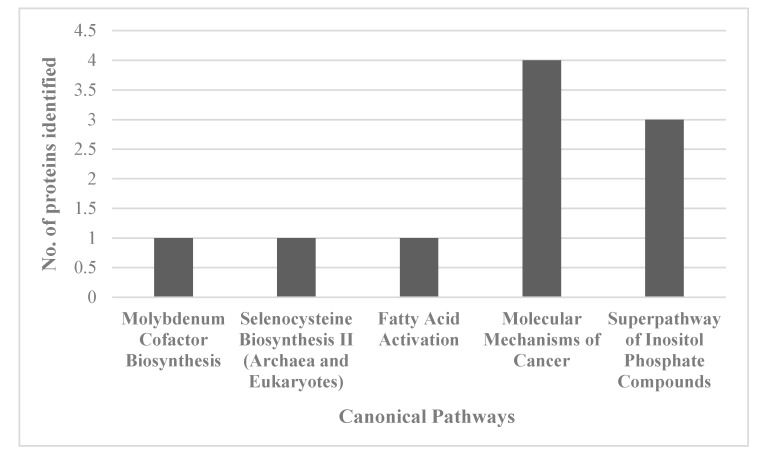
Graph of the number of proteins identified in the proteomics data set that map to the top 5 identified canonical pathways affected by AC-T treatment.

**Figure 10:**
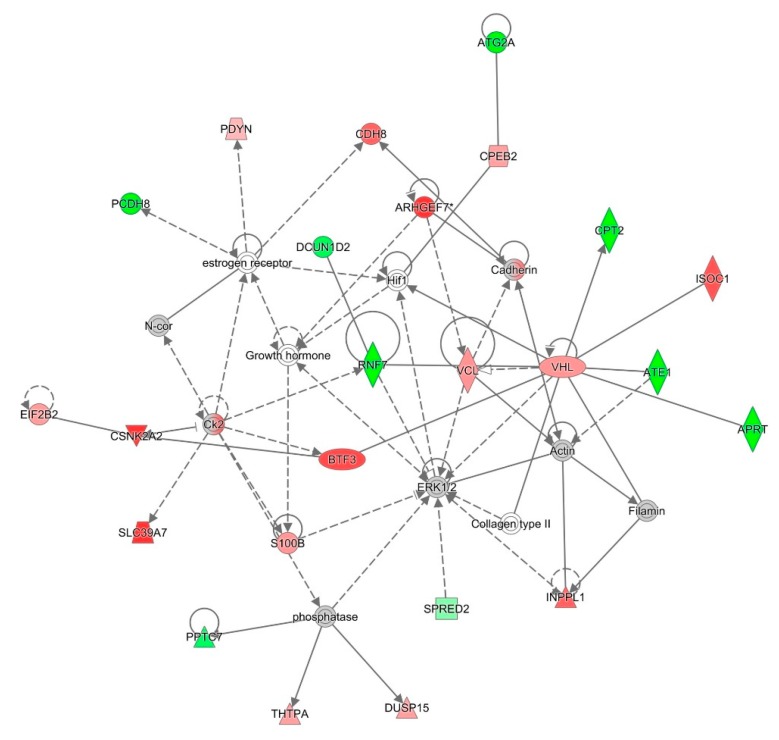

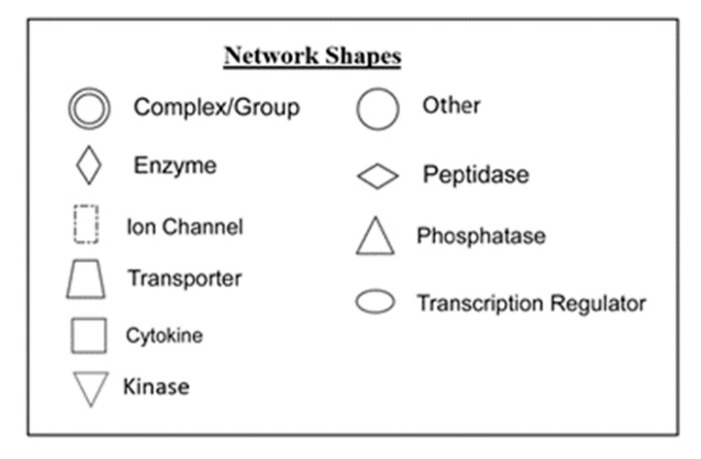
Graphic representation of mouse hippocampus protein network 1, identified by IPA as being affected by AC-T/MnBuOE treatment as compared to AC-T alone. Functions associated with the network include cardiovascular disease, cardiovascular system development and function, and cell morphology. The color of the node depicts differential expression. Red represents upregulated proteins. Green represents downregulated proteins. The intensity of the color denotes the degree of regulation where brighter colors are more regulated. Gray node color reflects proteins that were found in the data set but were insignificant.

**Figure 11:**
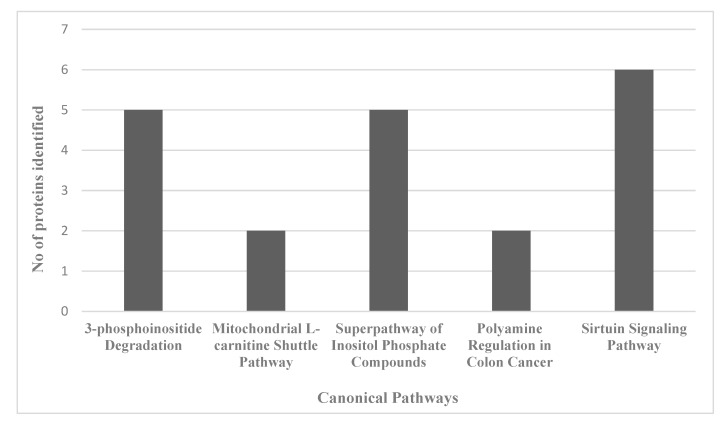
Graph of the number of proteins identified in the proteomics data set that map to the top 5 identified canonical pathways affected by AC-T/MnBuOE treatment as compared to AC-T treatment.

For an unbiased perspective of the molecular changes behind chemobrain, the datasets of differentially expressed proteins were uploaded to Ingenuity Pathway Analysis (IPA) application for pathway and network analysis. IPA bioinformatics identified the top protein networks associated with AC-T treatment with functions associated with neurological disease, cell morphology, behavior, and cell-to-cell signaling (Table 3 and Figure 8). IPA identified molybdenum cofactor and selenocysteine biosynthesis as top canonical pathways associated with AC-T treatment (Table 4 and Figure 9). Next, IPA ran the same analysis using the AC-T versus AC-T/MnBuOE differential protein dataset. The top protein networks associated with addition of MnBuOE included networks with functions associated with cell morphology, cell-to-cell signaling, nervous system development, and behavior (Table 5 and Figure 10). Top canonical pathways associated with MnBuOE treatment included the mitochondrial L-carnitine shuttle pathway, the super pathway of inositol phosphate compounds, and sirtuin signaling (Table 6 and Figure 11).

## 3. Discussion

We investigated the ability of MnBuOE to ameliorate the effects of AC-T chemotherapy on the hippocampus. Previously, it was demonstrated that female BALB/C mice receiving combination therapy (AC) showed deficits in spatial memory marked by a decrease in entries and movement in the target zone [26]. Our findings of behavioral deficits with the Morris water maze align with previous studies examining rodent models of cognitive decline after mono-or poly-chemotherapy treatment; however, this is the first time it was shown using the AC-T regimen. Another study noted the protective capacity of antioxidants, using the antioxidant astaxanthin, which reversed doxorubicin-induced cognitive impairment in mice [27]. In our case, the addition of MnBuOE to AC-T treated mice maintained intact spatial memory retention.

Dendritic branching alterations can disrupt synapse formation and/or stability, which ultimately can lead to neurological and cognitive disorders. Interestingly, chemotherapy drugs have been found to negatively impact dendritic branching. Chronic AC treatment significantly reduces total dendritic length and complexity in the hippocampus of female C57BL/6 mice [28]. We found that AC-T chemotherapy decreased dendritic length and complexity in the DG and CA1 regions of the hippocampus. MnBuOE treatment by itself did not affect dendritic length or spatial memory retention. The combination AC-T/MnBuOE maintained dendritic length in the DG and CA1 areas. 

Changes in the shape or size of the dendritic spines affect their stability and synaptic transmission [29]. Kang et al. found that AC treatment significantly decreased number of mushroom spines in the CA1 neurons of female black mice [28]. We did not see a change in the proportion of mushroom spines after AC-T treatment in the DG or CA1 neurons. Yet, MnBuOE treatment itself increased the number of mushroom spines which are considered the most stable type of spine [30,31]. Increasing the number of dendritic mitochondria or increasing their activity is known to enhance the number and plasticity of spines [32]. A plausible explanation is under normal redox circumstances, MnBuOE could contribute to the formation of mushroom spines by increasing mitochondrial health and/or activity since it accumulates in the mitochondria [20]. Further experiments would be needed to investigate this possibility.

We took a shot-gun proteomic approach to look at how AC-T treatment affected the hippocampus of adult female mice. Mitochondrial specific proteins were both up- and downregulated. Brain mitochondrial carrier protein 1 was found to be upregulated and serves to reduce mitochondrial membrane potential. Conversely, downregulation of the protein C19orf12 homolog is associated with response to oxidative stress. Another downregulated protein was an accessory subunit for NADH dehydrogenase or complex I of the respiratory chain. 

The canonical pathways significantly affected by AC-T treatment were molybdenum biosynthesis and selenocysteine biosynthesis which are both involved in redox reactions [33,34]. Fatty acid activation was also affected which can occur in the outer mitochondrial membrane. This indicates that our chemotherapy regimen affects multiple mitochondrial processes. Curiously, the super pathway of inositol phosphate compounds was affected. Despite it being a broad pathway, inositol phosphate compounds, especially inositol 1, 4, 5-trisphosphate (IP3), play an important role in internal calcium ion signaling. Dysregulation of the IP3 receptor pathway has been implicated in neurodegenerative disorders and could provide insight into the mechanisms behind chemobrain [35]. Network analysis revealed affected networks whose broad functions include behavior and cell morphology both of which we found to be affected by AC-T.

The next proteomic comparison involved the effects of MnBuOE on AC-T treated mice. Protein C19orf12 homolog was also found to be downregulated. We found that among the most significantly affected canonical pathways was again the super pathway of inositol phosphate compounds. We also saw the mitochondrial L-carnitine shuttle pathway affected. Doxorubicin has been shown to disrupt the carnitine system contributing to impaired energy metabolism [36] Chemotherapy can lead to cachexia involving perturbed energy balance and mitochondrial dysfunction [37]. We also saw the polyamine regulation in the colon cancer pathway as significantly affected. In regards to brain tissue, polyamines serve as modulators of NMDA (*N*-methyl-d-aspartate) and AMPA (α-amino-3-hydroxyl-5-methyl-4-isoxazole-propionate) glutamate receptors [38]. These receptors are key regulators of long-term potentiation. Sirtuin signaling was also found to be affected. Sirtuins are NAD+ dependent deacetylases that regulate cell responses to stress and are considered to play critical roles in cancer, aging, and neurodegenerative diseases [39,40]. Network analysis revealed the top network to have functions associated with the cardiovascular system and cell morphology. Both doxorubicin and cyclophosphamide have cardiotoxic side effects, so it is not surprising to see proteins associated with cardiovascular signaling. The other top networks also had broad functions associated with behavior. The proteomics analysis suggests mitochondrial dysfunction, oxidative stress, and energy metabolism as possible mechanisms behind chemobrain.

## 4. Materials and Methods 

### 4.1. Animals

Sixteen-week-old female C57Bl6/J wild-type mice (*n* = 48) were purchased from Jackson Laboratory (Bar Harbor, ME). The mice were housed either five or two mice per cage on constant 12:12 light-dark cycle. Food (Tekland Rodent Diet 8604, Envigo) and water were provided ad libitum. All experiments carried out were approved by the Institutional Animal Care and Use Committee at UAMS (number 3831, approval date 28 March 2018).

### 4.2. Chemotherapy Paradigm

**Figure 12:**
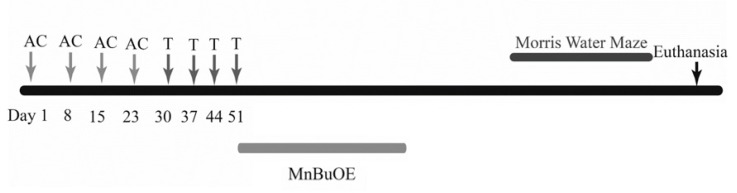
Graphical representation of experimental design.

The chemotherapeutic drugs were purchased from the UAMS Inpatient Pharmacy and stored according to manufacturer’s instructions. Doxorubicin (A) and cyclophosphamide (C) were diluted using sterile saline to make stock solutions. The mice received weekly intraperitoneal injections of saline (0.9% saline; *n* =12) or doxorubicin (2 mg/kg; *n* =12) and cyclophosphamide (50 mg/kg: *n* =12). The AC regimen was given over four weeks totaling four injections (Day 1, 8, 15, and 22) (Figure 12). The next cycle of chemotherapy included weekly intraperitoneal injections of paclitaxel (T) (5 mg/kg; *n* = 12) for 4 weeks. This constituted the AC-T regimen. MnBuOE (a kind gift from Dr. James Crapo, National Jewish Health, Denver, CO, 3 mg/kg; *n* =12) was then administered twice weekly subcutaneously for eight injections over 4 weeks. All animals were injected between 1400 h-1700 h.

Dosages were selected based on standard human chemotherapeutic doses. Patients receive four cycles of AC (doxorubicin 60 mg/m^2^ and cyclophosphamide 600 mg/m^2^) followed by four cycles of paclitaxel (175 mg/m^2^). To translate the clinical dose to animals, the body surface area normalization was used. Previous work by Flanigan et al. showed that doxorubicin (2 mg/kg) and cyclophosphamide (50 mg/kg) was sufficient to cause changes in hippocampal morphology without acute toxicity. The dosages of doxorubicin and paclitaxel translate to human equivalent doses that are below clinical dose. Paclitaxel was then chosen to be given at 5 mg/kg for a cumulative dose of 20 mg/kg, the maximum tolerated single dose for mice [41]. Cyclophosphamide was given at a clinically relevant dose.

### 4.3. Water Maze

The Morris water maze (MWM) was used to assess hippocampal-dependent spatial learning and memory [42]. A circular pool (133 cm diameter) was filled with opaque water (24 °C). The MWM is a 5-day task consisting of two sessions per day (2 h apart). Each session consisted of three trials, resulting in six trials daily. Trials were conducted with 10-min intertrial intervals. The mice were placed into the water facing the edge of the pool. Trials ended when the mouse located the platform. If a mouse failed to locate the platform within 60 s, they were led to the platform by the experimenter and required to sit on the platform for 10 s. Days 1 and 2 were training days using a visible platform. Days 3–5 consisted of hidden platform training which required the mice to rely on extra-maze cues. Following the second session on days 3–5, spatial memory retention was tested using a probe trial. The hidden platform was removed for this trial, and the mice were placed in the quadrant opposite to where the platform was at (target quadrant). The mice were allowed to swim for 60 s. Time spent in the target quadrant was compared to time spent in the non-target quadrants. Other measures of performance in the maze included average velocity and distance to the platform [43]. Ethovision video tracking system (Ethovision XT, Noldus Information Technology, Wageningen, Netherlands) was used to record all sessions.

### 4.4. Tissue Collection

The mice were sacrificed by cervical dislocation followed by decapitation. The brains were removed and dissected. The left hemisphere was dissected down to the olfactory bulb, cerebellum, hippocampus, prefrontal cortex, and cortex in cold phosphate buffered saline (1× PBS). The samples were snap frozen in liquid nitrogen followed by storage at −80 °C. The right hemisphere was stored in Golgi solution.

### 4.5. Golgi Staining

In order to look at dendrite morphology and spine dynamics, Golgi staining was used to randomly stain neurons in the hippocampus [44]. An adapted protocol was used with the reagents of the superGolgi kit (Bioenno Tech LLC, Santa Ana, CA, USA) [45]. Right hemispheres were impregnated with potassium dichromate solution for two weeks (*n* = 32) followed by 48 h in post-impregnation buffer. The brain was sectioned in the coronal plane at 200 µm thick slices using a vibratome. The slices were transferred into a 24-well plate for staining. The slices were washed in PBS-T (1× PBS, 0.3% Triton^TM^ X-100) before staining with ammonium hydroxide and washed in post-stain buffer. After another PBS-T wash, the slices were mounted on 1% gelatin-coated slides and left to dry overnight in Coplin jars. The next morning, the slides were dehydrated with ethanol, cleaned in xylene, and cover-slipped with Permount^TM^, Thermo Fisher Scientific^TM^ Inc., Waltham, MA, USA).

### 4.6. Dendritic Morphology

An experimenter blinded to treatment groups traced 5 randomly stained neurons per animal and averaged the values to include in the analysis. Specifically, neurons were traced in the DG and CA1 of the hippocampus. Sholl analysis was used to quantify the amount and distribution of the dendritic arbor [45,46]. A series of circles with increasing radii (10 µm intervals) were centered on the cell body [47]. Statistical analysis was done with GraphPad Prism 8 using a two-way mixed model with Sidak multiple comparisons. Branch-point analysis was used to quantify the complexity of the dendritic. First, the number of bifurcations (where one branch divides into two branches) is counted. Then, the branch-points were counted where first order points were defined where a primary branch splits into second-order branches and so on. Dendritic complexity analysis (DCI) = (sum (branch tip orders + number of branch tips)) X (total dendritic length/total number of primary dendrites). The Neuroexplorer component of the Neurolucida^®^ program (Microbright-field Science, Williston, VT, USA) was used for the analysis. For complexity, statistical analysis was done with an ordinary 1-way ANOVA with the Holm-Sidak correction for multiple comparisons.

### 4.7. Dendritic Spine Morphology

Dendritic spines were also analyzed using Golgi-stained brain sections featuring the hippocampus. The criteria for neurons chosen for analysis were the following: 1. non-truncated dendrites; 2. consistent Golgi staining along dendrites; 3. relative isolation from other neurons to avoid interference [48]. The neurons were traced using Neurolucida software version 11. Six to seven neurons per brain with 5 dendritic segments (20 nm long) per neuron were analyzed [47].

### 4.8. Proteomics

#### 4.8.1. Tissue Preparation

One day after the last MnBuOE injection, a small cohort (*n* = 4 per) were sacrificed for immediate protein analysis. The hippocampus was removed and placed in 400 µl of RIPA lysis buffer (10 mM Tris-Cl pH 8.0, 1 mM EDTA, 0.5 M EGTA, 1% Triton X-100, 0.1% sodium deoxycholate, 0.1% SDS, 140 mM NaCl). The tissue was homogenized on ice, incubated for 30 min on ice, and then centrifuged at 20,000× *g* for 10 min at 4 °C. The supernatant was transferred to a new microcentrifuge tube and stored at −80 °C until processing. The Compat-Able^TM^ Protein Assay Preparation Reagent Kit (Thermo Fisher Scientific Waltham, MA, USA) was used to eliminate EGTA as an interfering substance for the BCA Pierce TM BCA Protein Assay Kit (Thermo Fisher Scientific, Waltham, MA, USA). Protein was separated by SDS-PAGE using the 4–15% Criterion™ TGX™ Precast Midi Protein Gel, 18-well, 30 µL (Bio-Rad, Hercules, CA, USA) ran at 120V for 75 min. The gel was stained using Coomassie Blue staining (Bio-Rad, Hercules, CA, USA). The samples were then sent to the UAMS Proteomics Core for further processing for mass spectrometry.

#### 4.8.2. GeLC-MS/MS Analysis

Each SDS-PAGE gel lane was sectioned into 12 segments of equal volume. Each segment was subjected to in-gel trypsin digestion as follows. Gel slices were destained in 50% methanol (Thermo Fisher Scientific, Waltham, MA, USA), 100 mM ammonium bicarbonate (MilliporeSigma, St. Louis, MO, USA) followed by reduction in 10 mM Tris[2-carboxyethyl]phosphine (Thermo Fisher Scientific Waltham, MA, USA) and alkylation in 50 mM iodoacetamide (MilliporeSigma, St. Louis, MO, USA). Gel slices were then dehydrated in acetonitrile (Thermo Fisher Scientific Waltham, MA, USA), followed by addition of 100 ng porcine sequencing grade modified trypsin (Promega, Madison, WI, USA) in 100 mM ammonium bicarbonate (MilliporeSigma, St. Louis, MO, USA) and incubation at 37 °C for 12–16 h. Peptide products were then acidified in 0.1% formic acid (Thermo Fisher Scientific Waltham, MA, USA). Tryptic peptides were separated by reverse phase XSelect CSH C18 2.5 um resin (Waters, Milford, MA, USA) on an in-line 150 × 0.075 mm column using a nanoAcquity UPLC system (Waters, Milford, MA, USA). Peptides were eluted using a 30 min gradient from 97:3 to 67:33 buffer A: B ratio (buffer A = 0.1% formic acid, 0.5% acetonitrile; buffer B = 0.1% formic acid, 99.9% acetonitrile). Eluted peptides were ionized by electrospray (2.15 kV) followed by MS/MS analysis using higher-energy collisional dissociation (HCD) on an Orbitrap Fusion Tribrid mass spectrometer (Thermo Fisher Scientific Waltham, MA, USA) in top-speed data-dependent mode. MS data were acquired using the FTMS analyzer in profile mode at a resolution of 240,000 over a range of 375 to 1500 m/z. Following HCD activation, MS/MS data were acquired using the ion trap analyzer in centroid mode and normal mass range with precursor mass-dependent normalized collision energy between 28.0 and 31.0.

The mass spectrometry proteomics data have been deposited to the ProteomeXchange Consortium via the PRIDE [1] partner repository with the dataset identifier PXD017271 and 10.6019/PXD017271.

Project Name: Assessing the Effects of Redox Modifier MnTnBuOE-2-PyP 5+ on Cognition and Hippocampal Physiology Following Doxorubicin, Cyclophosphamide, and Paclitaxel Treatment. Project accession: PXD017271. Project DOI: 10.6019/PXD017271.

#### 4.8.3. Data Analysis

Proteins were identified and quantified by searching the UniprotKB database restricted to *Mus musculus* using MaxQuant (version 1.6.5.0, Max Planck Institute). The database search parameters included selecting the MS1 reporter type, trypsin digestion with up to two missed cleavages, fixed modifications for carbamidomethyl of cysteine, variable modifications for oxidation on methionine and acetyl on N-terminus, the precursor ion tolerance of 5 ppm for the first search and 3 ppm for the main search, and label-free quantitation with iBAQ normalized intensities. Peptide and protein identifications were accepted using the 1.0% false discovery rate identification threshold. Protein probabilities were assigned by the Protein Prophet algorithm [49]. 

MaxQuant iBAQ intensities for each sample were median-normalized so the medians were equal to the sample with the maximum median. Median-normalized iBAQ intensities were then imported into Perseus (version 1.6.1.3, Max Planck Institute) to perform log2 transformation and impute the missing values using a normal distribution with a width of 0.3 and a downshift of 2 standard deviations. The Linear Models for Microarray Data (limma) Bioconductor package was used to calculate differential expression among the experimental conditions using the lmFit and eBayes functions [49,50]. Proteins were considered to be significantly different with a fold change >2 and a false discovery rate adjusted *p*-value < 0.05. Differentially expressed proteins were analyzed using Ingenuity Pathway Analysis (IPA, QIAGEN Redwood City, www.qiagen.com/ingenuity) to identify pathways and networks. 

Visible- and hidden-platform water-maze learning curves were analyzed by two-way repeated-measures ANOVA. We used Holm’s correction to control for multiple comparisons, and separate analyses were conducted for the visible- and hidden-platform learning curves. For analysis of performance in the MWM probe trials, we used one-way ANOVAs with Holm’s post-hoc test, when appropriate. For measures of dendritic intersections, a mixed-factors ANOVA tested for the effects of chemotherapy (as the between-subjects variable) and distance from the cell body (Sholl radius, repeated measures variable). ANOVAs were followed by Holm’s post hoc tests when appropriate. 

## 5. Conclusions

We used adult female mice to measure the effects of AC-T chemotherapy on hippocampal morphology and spatial memory. We observed decreased spatial memory retention in the Morris water maze for AC-T treated animals as compared to controls. AC-T/MnBuOE was observed to have intact spatial memory retention. We also found alterations in dendritic morphology in the hippocampus of AC-T treated mice. AC-T/MnBuOE preserved dendritic morphology. MnBuOE treatment alone increased the number of mushroom spines. Proteomic network analysis revealed networks associated with cell morphology and behavior, in which we found deficits with AC-T treatment. Further work elucidating the contribution of oxidative stress to cognitive impairment after chemotherapy will help inform us of potential therapeutics such as the SOD mimetic, MnBuOE. Next, it is important to look at the effects of chemotherapy on cognition, dendritic morphology, mitochondrial dysfunction, and oxidative stress in tumor-bearing animals in order to see the effects of MnBuOE on cancer.

## Figures and Tables

**Table 1:** Top dysregulated proteins associated with AC-T treatment. A comparison of AC-T treated mice versus saline-treated mice identified 211 proteins as meeting statistical significance for differential expression. See Table S1 for a full list of proteins and Table S2 for specific peptide count information.

Protein	Description	Location in Cell	Type	Fold Change (log ratio)
Q8WUR0	Protein C19orf12 homolog	ER, Cytosol, Mitochondrion	Transmembrane	–5.53
P97291	Cadherin-8	cell membrane	Structural	–5.28
Q544H8	Zinc finger protein 161, isoform CRA_a	cytosol, nucleus, aggresome	Regulatory	–5.16
Q9CPU2	NADH dehydrogenase (ubiquinone) 1 beta subcomplex subunit 2	mitochondrion	Accessory	–4.96
F6RBX1	Target of myb-like protein 2 (Fragment)	NA	Other	–4.79
A0A0A0MQJ8	Brain mitochondrial carrier protein 1	Mitochondrion	Transporter	4.31
Q8BNZ1	t-SNARE coiled-coil homology domain-containing protein	Golgi apparatus	Membrane	4.51
A0A140LJ31	Putative lipoyltransferase 2 (Fragment)	Mitochondrion	Enzyme	4.80
I4DCY6	Sigma non-opioid intracellular receptor 1	ER	Transmembrane	5.03
Q3UJR8	Basic transcription factor 3	Cytosol	Regulatory	5.34

**Table 2:** Top dysregulated proteins associated with AC-T/MnBuOE treatment. A comparison of AC-T treated mice versus AC-T/MnBuOE treated mice identified 147 proteins as meeting statistical significance for differential expression. See Supplementary Table S1 for a full list of proteins and Table S3 for specific protein counts.

Protein	Description	Location in Cell	Type	Fold Change (log ratio)
Q9WTZ1	RING-box protein 2 (Rbx2)	Nucleus, cytoplasm	Enzyme	–4.84
Q8WUR0	Protein C19orf12 homolog	ER, Cytosol, Mitochondrion	Transmembrane	–4.08
Q8BNZ1	t-SNARE coiled-coil homology domain-containing protein	Golgi apparatus	Membrane	–4.03
Q9D4I9	RAB23, member RAS oncogene family, isoform CRA_a	Cytoskeleton, cytosol, plasma membrane	Enzyme	–3.92
A0A0R4J0X8	Rho guanine nucleotide exchange factor (GEF7), isoform CRA_a	Cytoplasm	Enzyme	4.56
A2BI12	PC4 and SFRS1-interacting protein	Nucleus	Regulatory	5.24
Q8BHC4	Dephospho-CoA kinase domain-containing protein	Mitochondrion	Enzyme	5.26
Q3U2K2	Uncharacterized protein (Fragment)	Membrane	Transmembrane	5.27
Q3U506	Uncharacterized protein	NA	NA	5.80

**Table 3:** Top five IPA protein networks associated with AC-T treatment.

Network Rank	Network Description
Network 1	Associated network functions: neurological disease, cell morphology, cell-to-cell signaling and interaction
	Number of “focus molecules” contained in the network: 22
	IPA p-score: 49
	Network proteins: ACSL4, APOA4, ARL1, Akt, Ampa Receptor, CACNG2, CDH8, CDK5RAP3, CFAP20, Creb, ERK, ERK1/2, Filamin, INPPL1, ISOC1, Insulin, Jnk, LDL, LRPAP1, MAP3K4, Mapk, NFkB (complex), P38 MAPK, PI4KB, PON2, PSEN1, Pka, RTF1, S100B, SPRED2, SYNGAP1, TCF4, TMCO1, VAMP2, VHL
Network 2	Associated network functions: cell death and survival, cancer, cell-to-cell signaling and interaction
	Number of “focus molecules” contained in the network: 16
	IPA p-score: 33
	Network proteins: ARHGAP17, ATP5MD, DUSP15, EGLN3, FANCD2, GPX3, GRB2, LIG1, LRRC40, LSM12, LSM4, LY6D, LYPLAL1, MLLT11, MRPS33, OSM, PDZD11, PIGK, PRAG1, PTEN, PTPN18, QRICH1, RAB30, RIOX1, SEPHS1, SHIP, TNF, TNFRSF10D, UAP1L1, UNC119B, WIPF2, YIF1B, ZBTB25, cerebroside 3-sulfate, glycosylphosphatidylinositol
Network 3	Associated network functions: behavior, nervous system development and function, cell-to-cell signaling and interaction
	Number of “focus molecules” contained in the network: 15
	IPA p-score: 30
	Network proteins: ANKS1B, APP, Atp5e, CASC4, CFDP1, COPS7B, Cops2, DCAF5, DCAKD, DYNLT1, ERP44, FBXO21, H2AFJ, ITM2C, KCNA4, KIAA0586, MOCS2, NFYB, PDCD7, PNMA8B, RABL3, RWDD2A, RWDD2B, SCN1A, SRP19, SRP68, SRP9, SUN5, SV2B, TLL1, TMEM189, UBC, UBL3, UQCRHL, WRNIP1
Network 4	Associated network functions: cellular development, cellular growth and proliferation, cell cycle
	Number of “focus molecules” contained in the network: 12
	IPA p-score: 23
	Network proteins: ARG1, ATG101, BOD1L1, CDC7, CDK2, CDK5R1, CHN2, CPNE3, ERBB2, GLCE, GRPEL1, HMGA2, LRRC4, MRPL55, MYL3, NF2, NRBP1, NTNG1, PLAC8, POLR3E, RB1, RBMS3, RMDN1, SAMHD1, SLC25A14, SPP1, TAX1BP3, TGFB1, TMEM94, TP53RK, TSC22D2, TTC9B, VSIG4, ammonia, dTTP
Network 5	Associated network functions: cell morphology, nervous system development and function, tissue morphology
	Number of “focus molecules” contained in the network: 10
	IPA p-score: 18
	Network proteins: AIG1, AP5Z1, BARX2, CHL1, Ck2, DBF4, DDX3Y, E2F1, EPGN, ESR1, FHL1, FOS, FUNDC2, FXYD6, GLP1R, Groucho, HOXA7, Hd-neuronal intranuclear inclusions, LEPROTL1, LMNA, Mta, N-cor, NUCKS1, PDCD5, PDLIM3, PSIP1, Rb-E2F transcription repression, SIN3A, SLC25A42, Sin3, TBL1XR1, TLE1, U2SURP, XK, ZFP36

IPA generates a non-directional network of interaction from overlaying focus molecules found in the experimental dataset with their Global Molecular Network. A proprietary algorithm generates the connectivity. IPA computes a p-score = −log10 (*p*-value); the *p*-value is calculated by Fisher’s exact test. The score looks at the fit of the supplied data set and a list of biological functions in the IPA Knowledge Base. The number of molecules in the network and network size are taken into account to assess the network’s relevance to the original list of proteins.

**Table 4:** Top five IPA-identified canonical pathways affected by AC-T treatment.

Pathway Name	*p*-Value	IPA Ratio
Molybdenum Cofactor Biosynthesis	0.01348963	0.25
Selenocysteine Biosynthesis II (Archaea and Eukaryotes)	0.01995262	0.167
Fatty Acid Activation	0.04265795	0.0769
Molecular Mechanisms of Cancer	0.04365158	0.0102
Superpathway of Inositol Phosphate Compounds	0.04570882	0.0127

The IPA ratio divides the number of molecules that meet criteria by the total number of pathway proteins listed in the IPA database. The *p*-value represents the probability of the ratio occurring by chance.

**Table 5:** Top five IPA protein networks associated with the comparison of AC-T vs. AC-T/MnBuOE treatments.

Network Rank	Network Description
Network 1	Associated network functions: cardiovascular disease, cardiovascular system development and function, cell morphology
	Number of “focus molecules” contained in the network: 24
	IPA p-score: 49
	Network proteins: APRT, ARHGEF7, ATE1, ATG2A, Actin, BTF3, CDH8, CPEB2, CPT2, CSNK2A2, Cadherin, Ck2, Collagen type II, DCUN1D2, DUSP15, EIF2B2, ERK1/2, Filamin, Growth hormone, Hif1, INPPL1, ISOC1, N-cor, PCDH8, PDYN, PPTC7, RNF7, S100B, SLC39A7, SPRED2, THTPA, VCL, VHL, estrogen receptor, phosphatase
Network 2	Associated network functions: cell-to-cell signaling and interaction, nervous system development and function, infectious diseases
	Number of “focus molecules” contained in the network: 24
	IPA p-score: 49
	Network proteins: 26s Proteasome, ANKS1B, APOA4, Akt, Ampa Receptor, CACNG2, COPS7B, DCAF5, DHRS7, ELMOD1, ERC1, ERP44, HDL, ITPR, IgG1, Ikb, MAP3K4, MHC Class II (complex), MYD88, NFkB (family), OSTF1, PDZD11, PSEN1, PSME1, Pro-inflammatory Cytokine, RBM8A, RILPL1, SERPINA1, SIGMAR1, SMG8, SYNGAP1, TBK1, TMEM263, TOM1L2, Ubiquitin
Network 3	Associated network functions: cell death and survival, skeletal and muscular disorders, behavior
	number of “focus molecules” contained in the network: 17
	IPA p-score: 31
	Network proteins: AGA, AK2, Ap1, CDK5RAP3, CTSS, Creb, F Actin, FCGBP, FSH, GAS2L1, GCLM, IgG, Igm, Immunoglobulin, LDL, Lh, MTA3, Mapk, Mek, NFkB (complex), PARP, PARP1, PDCD6, PGAM2, PI3K (complex), PIP4P2, PPAT, PRKAB2, RGS8, RTF1, TCR, TNFAIP8, Vegf, caspase, cytochrome C
Network 4	Associated network functions: embryonic development, organ development, organismal development
	Number of “focus molecules” contained in the network: 16
	IPA p-score: 28
	Network proteins: ADAM19, AP3S1, APRT, ATP5MD, Anti-inflammatory Cytokine, BOD1L1, CAMK2, CCDC85A, CDH4, CDH8, CLMN, CNRIP1, CPNE3, CREB1, DSC2, ESR2, FPR2, FUNDC2, GTF2F2, HABP2, IL4, ITPA, ITPK1, IgG4, JUNB, LMAN2, MOCS2, RAB30, RMDN1, SLC25A14, SPINT1, TGFB1, UBL3, YPEL5, ZBTB14
Network 5	Associated network functions: RNA post-transcriptional modification, cell cycle, connective tissue disorders
	Number of “focus molecules” contained in the network: 15
	IPA p-score: 26
	Network proteins: ALDH18A1, APEH, AQR, ARHGAP17, CDCA7L, CFAP300, CMTR1, COG1, CUL3, HNRNPUL2, INTS6, MLH1, MORC2, MRPS7, NTRK1, PABPN1, PAPSS1, POLR3B, PRPF6, PSIP1, RAMAC, RHOT1, RPAP1, SEPHS1, SLC25A23, SLFN11, SMNDC1, THOC2, TPRG1L, TRA2B, U2SURP, WDR90, WRNIP1, XAB2, XPNPEP3

**Table 6:** Top five IPA-identified canonical pathways affected by AC-T/MnBuOE treatment as compared to AC-T treatment alone.

Pathway Name	*p*-Value	IPA Ratio
3-phosphoinositide Degradation	0.00278	0.0323
Mitochondrial L-carnitine Shuttle Pathway	0.00484	0.118
Superpathway of Inositol Phosphate Compounds	0.00778	0.0253
Polyamine Regulation in Colon Cancer	0.00806	0.0909
Sirtuin Signaling Pathway	0.00965	0.0205

The IPA ratio divides the number of molecules that meet criteria by the total number of pathway proteins listed in the IPA database. The *p*-value represents the probability of the ratio occurring by chance.

## Supplementary Materials

Supplementary materials can be found at https://www.mdpi.com/1422-0067/21/5/1867/s1.

## Abbreviations

MDPI	Multidisciplinary Digital Publishing Institute
DOAJ	Directory of open access journals
TLA	Three letter acronym
LD	linear dichroism
AC-T	doxorubicin, cyclophosphamide, and paclitaxel
CA 1	Cornu Ammonis 1
DG	Dentate Gyrus
CICI	Chemotherapy Induced Cognitive Impairment
BC	Breast Cancer
ROS	Reactive Oxygen Species
BBB	Blood Brain Barrier
TNFα	tumor necrosis factor alpha
NOS	nitric oxide synthase
IPA	Ingenuity Pathway Analysis
NMDA	N-methyl-D-aspartate
AMPA	α-amino-3-hydroxy-5-methyl-4-isoxazolepropionic acid
